# Questions Regarding the Implementation of EU Mutagenesis Ruling in France

**DOI:** 10.3389/fpls.2020.584485

**Published:** 2020-09-11

**Authors:** Detlef Bartsch, Ulrich Ehlers, Frank Hartung, Jens Kahrmann, Georg Leggewie, Thorben Sprink, Ralf Wilhelm

**Affiliations:** ^1^ Department of Genetic Engineering and other Biotechnological Processes, Federal Office of Consumer Protection and Food Safety, Berlin, Germany; ^2^ Institute for Biosafety in Plant Biotechnology, Julius Kühn-Institute, Quedlinburg, Germany

**Keywords:** plant breeding regulation, mutagenesis techniques, Clearfield oilseed rape, risk coherence, European Commission (EC) decisions

## Abstract

The European Commission has asked EU Member States for comments on a French law notification demanding plant varieties produced with the help of *in vitro* mutagenesis have to be eliminated from the national catalog of approved varieties because of missing legal authorization deemed required by genetic engineering law. Primary target are herbicide-tolerant Clearfield oilseed rape varieties. The scientific reasoning is questionable, traceability is illusive, and law enforcement is likely to be impossible.

## Introduction

In reaction to the ruling of the European Court of Justice (ECJ) on mutagenesis (case C-528/16), the French Conseil d’État decided on a lawsuit brought in by various French farmer- and NGO-bodies concerning the national implementation of the mutagenesis exemption clause as given in Directive 2001/18/EC, Annex I B (1) in conjunction with recital 17. In its ruling, the Conseil d’État concluded that *in vitro* mutagenesis has not conventionally been used before 2001 in a number of applications and, hence, cannot be subsumed under the before mentioned exemption clause^1^. According to the court, plant varieties produced with the help of *in vitro* mutagenesis, therefore, have to be eliminated from the national catalog of approved varieties because of missing legal authorization deemed required by genetic engineering law. The European Commission has asked Member States for comments on this notification^2^. Hereinafter, both the historical timeframe of development and scientific application of *in vitro* mutagenesis in plant breeding will be briefly summarized. Finally, we highlight implications on traceability and law enforcement.

## Traditional Plant Breeding and Conventional Mutagenesis Techniques

Breeding is highly dependent on genetic diversity. Selection breeding uses diversity created through natural mutation, homologous recombination, and crossing over events created during each generation cycle when saving seeds of the best individuals for next year’s sowing. Since a hundred fifty years crossing based breeding tries to introduce new diversity by sexually combining varieties with variable traits. The beneficial combination of diverse genetic material of one species and the selection of suitable variants is the key to adapt crops to future agricultural needs. Next to selection and crossing, genetic diversity can be enhanced by creating a multitude of mutations using mutagenesis.

Conventional mutagenesis is applied mostly as physical mutagenesis by the help of irradiation. Seventy percent of the mutant varieties at the FAO/IAEA database (https://mvd.iaea.org/#!Home) were obtained *via* irradiation, the first one (tobacco, Chlorina F1) as early as 1928. Meanwhile more than 3,300 varieties are registered in this database. As these are voluntary registrations, even more mutagenized varieties and crossings thereof might be traded at present. Ionizing and non-ionizing irradiation causes structural changes of genomes with deletions, insertions, chromosomal rearrangements, and areas of chemically damaged DNA. Techniques used are α- and β-particle bombardment and irradiation with ions. Furthermore, X-rays, γ-irradiation, cosmic radiation, neutron, and UV radiation are used, the latter causing rather mild effects because of low tissue penetration energy.

Chemicals can induce mutations as well. They may either chemically interact with the bases of DNA like alkylating chemicals such as ethyl methanesulfonate (EMS), methyl nitrosourea (MNU), or methyl methanesulfonate (MMS). Transferring a methyl or ethyl group to a base of the DNA often causes transversions. Other chemicals used are antibiotics, analogs of bases or chemicals that intercalate with DNA like the dye ethidium bromide. Often chemical mutagenesis results in fewer and smaller mutations than irradiation. In some cases, no extra chemical is used but mutagenesis is achieved by disturbance of genetic processes due to components of a culture medium used in tissue culture and other artificial growth conditions (somaclonal variation).

## Risk Coherence For *In Vivo* and *In Vitro* Mutagenesis

Physical and chemical mutagenesis techniques can be applied *in vivo* and *in vitro*. However, Directive 2001/18/EC does not differentiate between “*in vivo*” and “*in vitro*” mutagenesis nor is there such a differentiation within the ruling of the ECJ on case C-528/16. Paragraph 23 of said ruling, in which both words are mentioned, merely summarizes the case and legal arguments as presented by the French Conseil d’Etat as the referring court.

Scientifically, the term *in vivo* means processes that happen within the living organism. Mutagenesis applied to entire plants or parts thereof was and is frequently used with plants that are sexually propagated, mostly annual plants. Seeds are treated for various time spans with radiation or chemicals and germinating plants are selected for useful traits. Annual plants constitute the majority of mutagenized varieties registered. Vegetative propagated plants are mutagenized as well by exposing plantlets, tubers, or bulbs to mutagenizing agents.

*In vitro*, in contrast, describes processes in a rather artificial environment and in general outside the living organism. In plant breeding, however, it is possible to submit entire plants, plantlets, and parts of plants to growth in closed environments such as jars or test tubes on artificial nutrient medium and under sterile conditions. These plant parts can be cultivated to form lumps of undifferentiated cells (calli) or even single cell cultures which are used to restore entire plants through special nutrient and hormone treatments. There is a clear genetic continuum from a single plant cell to an entire plant due to the pluripotency in plants. It is, therefore, not possible, to establish a clear cut borderline between *in vivo* and *in vitro* in the sense of *in vivo* meaning entire plants or plant parts (seeds) and *in vitro* meaning just single cells in artificial environments.

In general, the mutagenizing agent determines what kind of DNA-damage is created. There are no data supporting the idea that the spatial status of the affected cell (part of a callus, within a cell suspension, part of a plantlet or within a plant outside an artificial environment) may have any influence on the molecular mode of action of a mutagenizing agent on it. Ionizing irradiation creates a mixture of single strand and double strand breakage as well as chemical destruction of the bases. Chemical treatment such as EMS application modifies bases, e.g., *via* alkylation or interstrand crosslinks, other agents, e.g., *via* methylation. Which repair system is used for repair, again, is not triggered by the spatial status of the mutagenized cell but by the kind of damage. Chemically damaged bases may be repaired by base excision repair when alkylated or by nucleotide excision repair when bulky lesion occur, single or double strand DNA breaks are most often repaired by non-homologous end joining or homology dependent repair (for a recent review, see Chatterjee and Walker, 2017). Only if the genetic background of cells is different (e.g., repair deficient cells) or during meiosis, divergences in the usage of the different repair systems might be visible.

Another aspect is that any mutagenesis approach aims at obtaining a genetically homogenous organism. All cells of such an organism shall be identically mutated. The origin of such an organism is mostly a single cell that passed on the mutation obtained to all offspring cells constituting eventually the entire organism. For the resulting organism, it does not matter whether this “successful” cell was part of a callus, a single cell culture or a plantlet cultivated in an artificial environment.

It follows that in terms of risk, there is no scientific reasoning to apply a different regulatory regime to plants created by *in vitro* methods compared to those created by *in vivo* techniques of mutagenesis. Meanwhile, various scientific bodies issued statements that agreed on the fact that there are no biochemical differences identifiable between mutations created by *in vivo* or *in vitro* mutagenesis nor are there any differences in the phenotypes created (Haut Conseil des Biotechnologies, 2020).

## Historical Use of *In Vitro* Mutagenesis Techniques

Mutagenesis has been applied for *in vitro* cultivated plant parts (since 1960, Roest and Bokelman, 1980), reproductive organs and isolated embryos (Novak et al., 1985), anther cultures (Nitsch, 1969), isolated microspores and pollen (Nitsch, 1969; Mondeil, 1974), plant cell and protoplast suspension cultures (Eriksson, 1967; Carlson, 1970) and *in vitro* cultivated entire plants (Nitsch, 1969). Protoplast and cell suspension cultures as well as microspores and pollen cultures represent single plant cells objected to mutagenesis in the sense of the ruling of the French Conseil d’État.

These techniques have been used widely to produce varieties being marketed before 2001: The mutant variety database of IAEA (https://mvd.iaea.org/) lists, e.g., the following varieties, which originate from plant cells: Potato, somaclonal Jagakids Purple (1994), and White Baron (1997) from protoplast cultures, and rice, somaclonal Ohita 3 Gouand (1997), and Yume-Kaori (1993) from protoplasts. Ahloowalia et al. (2004) lists 24 canola varieties originating from mutagenized microspores. In fact, imidazoline-tolerant canola was developed by chemical mutagenesis of microspore cultures produced from canola variety Topas in 1989 and a high-breed version of the mutagenized canola was marketed as “Smart Canola” as early as 1995 (Tan et al., 2005). If one adds to this list mutagenesis of *in vitro* cultures using calli, plantlets, and other *in vitro* cultivated explant organs there are examples for applications before 2001 in crops such as maize, barley, wheat, sugar beet, rye, sweet potato, fruits like pear, apple, cherry, wine grape, vegetables such as chili, tomato, pepper, asparagus, cauliflower, onions, and ornamentals such as chrysanthemum as well. In summary, *in vitro* mutagenesis was developed and applied regularly to various crops long before 2001 and therefore resulting varieties can be subsumed under the exemption clause in Directive 2001/18/EC, Annex I B (1).

## No Retrospective Traceability of Traded Varieties

*In vitro* mutagenesis, so far, was not subjected to any regulation as it was considered exempted in Directive 2001/18/EC (as well as in its preceding Directive 90/220/EEC). For the early years of plant breeding, a GMO regulation did not even exist. There is no registration of any mutagenesis technique applied when varieties, e.g., in Germany, are authorized by the Federal Plant Variety Office to enter the national variety catalog. As there were no regulatory requirements to keep protocols on whether or how exactly mutagenesis was applied, for most varieties, this information cannot be retrieved anymore. This holds especially for gene pool sources developed by breeding bodies that do not exist anymore. Due to the so called breeders’ privilege, any variety can be used by a breeder as a gene pool to develop new varieties. To restore pedigrees back to the 60s or 70s for identifying any input of genetic material subjected to *in vitro* mutagenesis is illusive. Also, international trading and exchange of breeding material limits any attempts for backtracking the spreading of such treated genetic material within present varieties. From a technical point of view, it appears nearly impossible to assign retrospectively any random mutation to an *in vitro* or *in vivo* mutagenesis technique, and thus, retrospective traceability is not possible.

## Actionable Recommendations

The ruling of the Conseil d’État on *in vitro* mutagenesis is difficult to reconcile on the basis of scientific facts and the history of mutagenesis in plant breeding. Traceability as pre-requisite for law enforcement will not be possible retrospectively in most cases, and there is biologically no reason to assume any difference in risk between *in vitro* and *in vivo* mutagenesis techniques. The proposed French notion of *in vitro* mutagenesis will put the French ruling into contradiction with the exemption clause in conjunction with recital 17 as given in Directive 2001/18/EC.

## Author Contributions

The authors contributed in equal parts to the manuscript.

## Conflict of Interest

The authors declare that the research was conducted in the absence of any commercial or financial relationships that could be construed as a potential conflict of interest.

## Footnotes

1 Conseil d’Etat Decision No. 388649, ECLI: FR: CECHR: 2020: 388649.20200207, https://beta.legifrance.gouv.fr/ceta/id/CETATEXT000041569364, accessed 11 August 2020.

2
https://ec.europa.eu/growth/tools-databases/tris/en/search/?trisaction=search.detail&year=2020&num=280, accessed 11. August 2020.
